# RNA modifications in radiotherapy resistance and radiosensitization: epitranscriptomic regulation of tumor response to radiation

**DOI:** 10.3389/fcell.2026.1895114

**Published:** 2026-07-10

**Authors:** Hongran Qin, Shuqiang Yang, Jiawei He, Meijia Zhao, Luqian Zhao, Jingjing Wang, Xiulin Jiang, Xin Xu, Xiaowen Chen

**Affiliations:** 1 Department of Nuclear Radiation, Shanghai Pulmonary Hospital, School of Medicine, Tongji University, Shanghai, China; 2 College of Life Science, University of Chinese Academy of Sciences, Beijing, China

**Keywords:** DNA damage repair, epitranscriptomics, ferroptosis, radioresistance, radiosensitization, radiotherapy, RNA modifications, tumor microenvironment

## Abstract

Radiotherapy (RT) remains a major treatment for solid tumors, but durable tumor control is frequently limited by adaptive DNA repair, altered cell-death thresholds, cancer stemness, metabolic plasticity, and immune escape. RNA modifications have recently emerged as rapid post-transcriptional regulators that enable tumor, stromal, and immune cells to remodel these programs after irradiation. This Mini Review emphasizes three major themes. First, N^6^-methyladenosine (m^6^A) is the best-characterized RNA modification in RT response, with METTL3/METTL14, FTO, ALKBH5, YTH-domain readers, and IGF2BP proteins regulating DNA repair, apoptosis, ferroptosis, stemness, metabolism, and immune checkpoints in a highly context-dependent manner. Second, non-m^6^A modifications, including 5-methylcytosine (m^5^C), N^4^-acetylcytidine (ac^4^C), 7-methylguanosine (m^7^G), and A-to-I RNA editing, are increasingly linked to homologous recombination, metabolic adaptation, innate immune sensing, and immune evasion, although their RT-specific evidence remains limited and should be viewed as emerging rather than established. Third, therapeutic targeting of RNA-modifying enzymes may improve radiosensitization only when guided by tumor type, cellular context, RT dose and fractionation schedule, predictive biomarkers, and normal-tissue safety. Accordingly, we organize current evidence around RNA-modification machinery, tumor-intrinsic mechanisms of radioresistance, immune microenvironment remodeling, and barriers to clinical translation. We further highlight the need to move beyond single-axis models toward dynamic, spatial, and clinically validated analyses of RNA modification networks during fractionated RT.

## 1 Introduction

Radiotherapy (RT) is used in approximately 50%–60% of patients with cancer and remains central to definitive, adjuvant, and palliative treatment strategies (Giraud et al., 2025; Li et al., 2025a). Modern approaches, including intensity-modulated RT, stereotactic body RT, proton therapy, and FLASH RT, have improved dose conformity and broadened the biological scope of RT (Li et al., 2025a; Vozenin et al., 2022; Tang et al., 2024). In addition to inducing DNA damage, RT promotes oxidative stress, tumor-antigen release, innate immune sensing, and remodeling of the tumor microenvironment. These effects create opportunities for combination therapy but also impose strong selective pressure on tumor cells.

**FIGURE 1:**
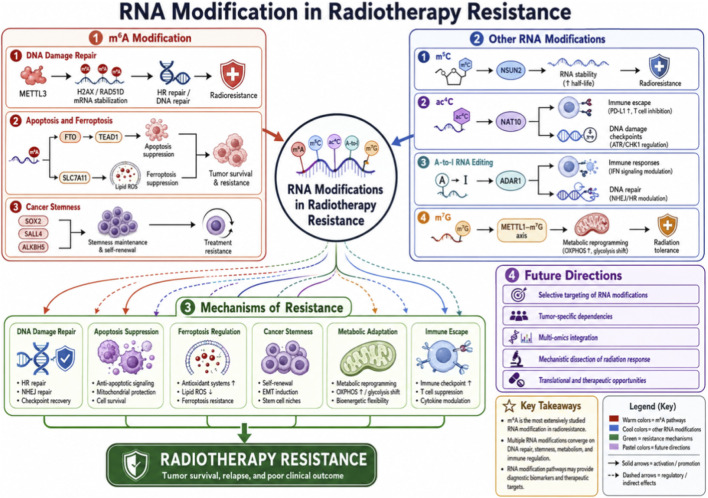
RNA modifications in radiotherapy resistance. RNA modifications contribute to radiotherapy resistance through multiple tumor-intrinsic mechanisms. m^6^A modification regulates DNA damage repair, apoptosis, ferroptosis, and cancer stemness by modulating the stability, translation, or degradation of key transcripts. Non-m^6^A modifications, including m^5^C, ac^4^C, A-to-I RNA editing, and m^7^G, also participate in RNA stability, immune escape, DNA damage checkpoints, and metabolic reprogramming. The figure highlights that different RNA modification pathways converge on DNA repair, cell-death suppression, stemness, metabolism, and immune escape, ultimately promoting radiotherapy resistance.

Radioresistance arises when tumor cells and their surrounding microenvironment adapt to radiation-induced stress. Major mechanisms include enhanced DNA damage repair, checkpoint recovery, suppression of apoptosis or ferroptosis, maintenance of cancer stemness, metabolic reprogramming, hypoxia, and immune escape (Nguyen et al., 2019; Wu et al., 2023). These processes are not independent. For example, metabolic adaptation can raise antioxidant capacity, stem-like cells often show stronger DNA repair, and immune suppression can convert radiation-induced inflammation into tumor tolerance. A streamlined model of these interconnected mechanisms is shown in Figure 1.

Epitranscriptomics has emerged as an important regulatory layer in cancer biology and therapy response. More than 170 RNA modifications have been identified, among which m^6^A, m^5^C, ac4C, m^7^G, and A-to-I RNA editing have attracted particular attention (Roundtree et al., 2017; Chen et al., 2024; Zhang L. et al., 2026). By affecting RNA splicing, export, stability, translation, decay, and innate immune sensing, these modifications can rapidly reshape gene-expression programs under therapeutic stress. This review focuses on how RNA modifications regulate RT response, avoiding repeated study-by-study descriptions and instead organizing the field around modification machinery, tumor-intrinsic resistance mechanisms, immune microenvironment remodeling, and translational challenges.

## 2 Core RNA modification machineries relevant to RT response

RNA modifications are generally controlled by writers that install marks, erasers that remove them, and readers that interpret them (Roundtree et al., 2017). The biological outcome depends not only on the modification type but also on the target transcript, cell type, reader availability, RT dose and timing, and the balance between tumor and normal tissue responses. Figure 1 summarizes how these machineries converge on major radioresistance phenotypes.

### 2.1 m^6^A machinery

m^6^A is the most abundant internal modification in eukaryotic mRNA and the best-studied RNA modification in RT biology (An and Duan, 2022). It is mainly installed by the METTL3-METTL14-WTAP writer complex, removed by FTO and ALKBH5, and interpreted by YTH-domain proteins, IGF2BP proteins, and HNRNP family members (Roundtree et al., 2017; An and Duan, 2022). In cancer, m^6^A controls transcripts involved in proliferation, DNA damage repair, stemness, metabolism, and immune escape (An and Duan, 2022; Wen et al., 2025; Wan et al., 2022). Its effects are highly context dependent: the same writer or reader may promote resistance in one tumor setting but enhance radiosensitivity or normal-tissue protection in another.

### 2.2 Non-m^6^A machineries

m^5^C is catalyzed mainly by NSUN family enzymes and DNMT2/TRDMT1 and can regulate RNA stability, export, and translation (Sun et al., 2023; Li and Huang, 2024; Chellamuthu and Gray, 2020; Zhou et al., 2026). In RT-related studies, NSUN2/NSUN6 and the m^5^C reader ALYREF have been linked to circRNA stability, homologous recombination, and ferroptosis resistance (Ren et al., 2026; Hu et al., 2026; Zheng et al., 2026; Zhang J. et al., 2026). Ac4C is primarily installed by NAT10, which enhances mRNA stability and translation but also has protein-acetylation functions relevant to checkpoints and metabolism (Xie L. et al., 2023; Gu et al., 2024; Zou et al., 2024; Liu et al., 2020). A-to-I RNA editing is catalyzed by ADAR enzymes, especially ADAR1, and can suppress dsRNA sensing, type I interferon responses, and antitumor immunity while also supporting DNA repair pathways (Song et al., 2022; Liang et al., 2024; Zhang et al., 2024) (Gan and Chen, 2025; Ishizuka et al., 2019; Tian et al., 2025). m^7^G is mainly mediated by the METTL1-WDR4 complex and can affect tRNA stability, codon-dependent translation, and selected mRNA programs (Orellana et al., 2021; Xiao et al., 2025).

Compared with m^6^A, the roles of m^5^C, ac^4^C, m^7^G, and A-to-I RNA editing in RT response should currently be viewed as emerging rather than established mechanisms. Most available studies focus on one tumor type, one RNA-modifying enzyme, or one downstream target axis, such as NSUN2-related DNA repair, NAT10-mediated ac^4^C regulation of KPNB1/PD-L1 localization, METTL1-dependent glycolytic adaptation, or ADAR1-associated DNA repair and immune suppression. These findings provide important proof-of-concept evidence, but they are not yet sufficient to support broad general conclusions across cancer types or RT settings. In particular, it remains unclear whether these modifications are globally and dynamically altered after irradiation, whether their effects are dose- or fractionation-dependent, and whether they act primarily through modified RNA targets or through modification-independent functions of their writer or editing enzymes. Therefore, non-m^6^A RNA modifications are best framed as promising but still incompletely defined layers of epitranscriptomic regulation in RT response. The major RNA modifications and their core regulatory machinery involved in RT response are summarized in Table 1.

**TABLE 1:** Major RNA modifications, core regulatory machinery, and RT-related biological processes.

Modification	Main machinery	RT-related processes emphasized in current literature	Ref
m^6^A	METTL3/METTL14/WTAP; FTO; ALKBH5; YTHDF/YTHDC, IGF2BP and HNRNP readers	DNA repair, apoptosis, ferroptosis, stemness, immune checkpoints, antigen presentation	(An and Duan, 2022; Wen et al., 2025; Wan et al., 2022) (Xiang et al., 2017) (Xu et al., 2023) (Du et al., 2023; Shi et al., 2024) (Kowalski-Chauvel et al., 2020; Zhou et al., 2025) (Yang et al., 2021)
m^5^C	NSUN family enzymes, DNMT2/TRDMT1, ALYREF	RNA stability/export, homologous recombination, circRNA stability, ferroptosis resistance	(Ren et al., 2026; Hu et al., 2026) (Sun et al., 2023) (Li and Huang, 2024) (Chellamuthu and Gray, 2020; Zhou et al., 2026)
ac4C	NAT10	mRNA stability/translation, PD-L1 nuclear transport, checkpoint control, metabolism and immune escape	(Xie et al., 2023a) (Gu et al., 2024; Gan and Chen, 2025) (Ishizuka et al., 2019) (Tian et al., 2025)
A-to-I editing	ADAR family, especially ADAR1	dsRNA sensing, type I interferon signaling, immune checkpoint response, Rad18-associated DNA repair	(Liang et al., 2024) (Zhang et al., 2024) (Gan and Chen, 2025) (Ishizuka et al., 2019) (Tian et al., 2025)
m^7^G	METTL1-WDR4	tRNA/mRNA regulation, codon-dependent translation, glycolytic adaptation and stress tolerance	(Sun et al., 2023) (Orellana et al., 2021; Xiao et al., 2025)

## 3 Tumor-intrinsic mechanisms of RNA modification-mediated radioresistance

Rather than treating each modification in isolation, the major RT-relevant functions can be organized into four interconnected themes: DNA damage repair, cell-death regulation, cancer stemness, and metabolic adaptation. This thematic view reduces redundancy and helps clarify why different RNA-modifying factors may produce opposite phenotypes across tumor types. To provide a concise overview of the current evidence, representative RNA modification-related mechanisms involved in RT response are summarized in Table 2.

**TABLE 2:** Representative RNA modification-related mechanisms in RT response.

Theme	Representative axes retained in the main text	Main implication	Ref
DNA repair	METTL3-H2AX; YTHDF3-RAD51D; METTL3-LNCAROD-PARP1; ALKBH5-CHK1/RAD51; NSUN2/NSUN6-RAD51/NDRG1; ADAR1-Rad18	RNA modifications often increase repair-transcript stability/translation or checkpoint recovery, but normal tissue repair must be considered	(Li and Huang, 2024; Xu et al., 2023) (Du et al., 2023; Shi et al., 2024) (Kowalski-Chauvel et al., 2020; Zhou et al., 2025)
Cell death and ferroptosis	YTHDF2-MYC; METTL3/IGF2BP2-TEAD1; METTL3-SLC7A11; FTO-OTUB1; METTL14-ACSL4; YTHDF2-SREBF1	Net radiosensitivity depends on the balance between pro- and anti-ferroptotic target transcripts	(Wang et al., 2024) (Fang et al., 2025a; Dai et al., 2025; Zhang et al., 2025a) (Huang et al., 2023) (Wang et al., 2023a; Dai et al., 2026)
Stemness and metabolism	METTL3-SOX2; FTO-RAD51/VEGFA; METTL3-SALL4-Wnt; WTAP-Bcl-2; METTL1-PFKFB3; NAT10/GSDMC-metabolic programs	RNA modifications couple stemness, repair, antioxidant defense, and metabolic plasticity	(Ren et al., 2026; Hu et al., 2026; Visvanathan et al., 2018; Zhang et al., 2025b; Huang et al., 2024; Wang et al., 2023b)
Immune regulation	ALKBH5/YTHDF2-HMGB1; YTHDF1-antigen presentation; YTHDF2-DC cross-presentation; METTL3/METTL14/ALKBH5-PD-L1/macrophages; NAT10-KPNB1-PD-L1	Cell-type and reader-dependent effects determine whether RT induces immune activation or immune escape (Wang et al., 2023b; Chatterjee and Walker, 2017; Yu et al., 2024; Lin et al., 2024; Shen et al., 2025; Tang et al., 2023; Biserova et al., 2021; Chen et al., 2023)	(Zhu et al., 2025; Chen et al., 2021) (Jhunjhunwala et al., 2021) (Lin et al., 2023) (Chen et al., 2026; Fang et al., 2025b; Zhang et al., 2026c) (You et al., 2022)

### 3.1 DNA damage repair and checkpoint recovery

DNA double-strand breaks are a central lesion induced by RT, and repair capacity strongly influences radiosensitivity (Chatterjee and Walker, 2017). m^6^A can support DNA repair at several levels. UV-induced DNA damage rapidly increases RNA m^6^A near damaged sites, where METTL3 catalytic activity facilitates recruitment of DNA polymerase κ (Xiang et al., 2017). In tumor RT models, METTL3 promotes H2AX mRNA stability and carbon-ion radioresistance in non-small cell lung cancer (Xu et al., 2023), while the HNF1α-YTHDF3 axis enhances m^6^A-dependent RAD51D translation and homologous recombination in cervical cancer (Du et al., 2023). m^6^A can also act through non-coding RNA: METTL3-stabilized LNCAROD protects PARP1 from ubiquitin-mediated degradation in esophageal squamous cell carcinoma (Shi et al., 2024). Demethylases and non-m^6^A writers also contribute to repair adaptation. In glioblastoma stem-like cells, the MST4-USP14-ALKBH5 axis maintains CHK1, RAD51, invasiveness, and radioresistance (Kowalski-Chauvel et al., 2020; Zhou et al., 2025). m^5^C-related NSUN2 and NSUN6 promote repair-associated radioresistance through TP53/RAD51 and NDRG1-dependent mechanisms, respectively (Zheng et al., 2026; Yu et al., 2024). ADAR1 supports non-small cell lung cancer radioresistance partly through Rad18 (Tian et al., 2025). NAT10 can regulate DNA damage responses through MORC2 acetylation, indicating that writer proteins may affect RT response through RNA modification-independent activities (Liu et al., 2020).

Collectively, current evidence suggests that RNA modifications do not simply enhance DNA repair globally, but rather reshape the repair capacity of irradiated tumor cells by selectively stabilizing or promoting the translation of key repair-related transcripts. A useful conceptual model is that RNA modifications function as rapid post-transcriptional “repair rheostats” under radiation stress. In this model, writers, erasers, and readers adjust the abundance or translational efficiency of DNA damage response factors according to tumor type, repair pathway dependence, and microenvironmental pressure. However, several questions remain unresolved. First, it is still unclear whether radiation-induced RNA modification changes are primary drivers of DNA repair activation or secondary adaptive responses to DNA damage. Second, most studies focus on single targets such as RAD51, PARP1, or H2AX, but few have examined whether multiple repair transcripts are coordinated by the same RNA modification program. Third, the extent to which these pathways are tumor-specific, rather than shared with normal tissue repair, remains a major concern for therapeutic targeting.

### 3.2 Cell-death thresholds: apoptosis, pyroptosis, and ferroptosis

Radiation efficacy depends not only on DNA damage but also on whether damaged cells undergo death. m^6^A participates in apoptosis after radiation-related stress: repeated UV irradiation changes the m^6^A landscape in HaCaT cells, and FTO overexpression suppresses apoptosis (Lin et al., 2024). In rectal cancer, YTHDF2 promotes degradation of methylated MYC mRNA, activates Hippo pathway signaling, and enhances radiation-induced apoptosis and G2/M arrest (Fang Y. et al., 2025). Conversely, m^6^A may contribute to normal tissue injury, as the METTL3/IGF2BP2-TEAD1-STING-NLRP3 axis promotes inflammatory liver injury after irradiation (Wang et al., 2024).

Ferroptosis is a particularly important and context-dependent node. The m^6^A-SLC7A11 axis often suppresses ferroptosis and promotes radioresistance in nasopharyngeal carcinoma and hepatocellular carcinoma (Dai et al., 2025; Zhang C. et al., 2025). FTO can reduce m^6^A on OTUB1 mRNA to reinforce anti-ferroptotic radioresistance, and FTO inhibition can cooperate with ferroptosis induction (Huang et al., 2023). KIAA1429-mediated m^6^A regulation of SLC7A11 further supports ferroptosis resistance in hepatocellular carcinoma (Wang H. et al., 2023). However, m^6^A can also promote ferroptosis: METTL14-mediated modification of ACSL4 mRNA enhances radiation-induced ferroptosis in esophageal squamous cell carcinoma, whereas YTHDF2-mediated stabilization of SREBF1 reduces lipid peroxidation and promotes radioresistance in anaplastic thyroid cancer (Dai et al., 2026). m^5^C regulation of DHODH also suggests a link between non-m^6^A marks and GPX4-independent ferroptosis resistance (Zhang J. et al., 2026).

The apparently opposite effects of m^6^A on ferroptosis may be explained by the identity and functional hierarchy of its target transcripts. Rather than being intrinsically pro- or anti-ferroptotic, m^6^A appears to determine the net ferroptotic outcome by shifting the balance among antioxidant defense, lipid peroxidation, iron metabolism, and membrane remodeling pathways. For example, m^6^A-dependent maintenance of SLC7A11, OTUB1, or SREBF1 tends to suppress lipid peroxidation and promote radioresistance, whereas m^6^A-mediated regulation of pro-ferroptotic targets such as ACSL4 may enhance ferroptosis and radiosensitivity (Dai et al., 2025; Shen et al., 2025; Tang et al., 2023). Therefore, the final effect of m^6^A on radiation response may depend on which transcript group dominates in a given tumor context. Future studies should move beyond single-gene models and define pathway-level m^6^A signatures that predict whether irradiated cells will undergo ferroptosis, apoptosis, senescence, or survival. It also remains important to determine whether combining RT with ferroptosis inducers can selectively exploit RNA modification-dependent vulnerabilities without increasing normal tissue injury.

### 3.3 Cancer stemness and metabolic adaptation

Cancer stem-like cells are enriched after treatment and often display strong DNA repair, anti-apoptotic signaling, and metabolic flexibility (Biserova et al., 2021). m^6^A maintains stemness in several RT models. METTL3 stabilizes SOX2 mRNA in glioblastoma stem-like cells and supports neurosphere formation, DNA repair, and radioresistance (Visvanathan et al., 2018). ALKBH5 and its upstream stabilizing axis preserve homologous recombination and resistant glioblastoma stem-like phenotypes (Kowalski-Chauvel et al., 2020; Zhou et al., 2025). FTO inhibition reduces glioblastoma stemness, RAD51/VEGFA expression, and radioresistance in orthotopic models (Zhang J. et al., 2025). In oral squamous cell carcinoma, METTL3 activates the SALL4-Wnt/β-catenin pathway in CD44^+^ stem-like cells (Huang et al., 2024), while WTAP-related m^6^A regulation of Bcl-2 contributes to NRP1-mediated breast cancer stemness and radioresistance (Wang Y. et al., 2023).

Metabolic adaptation is another convergent mechanism. RNA modifications can tune glycolysis, lipid metabolism, and mitochondrial fitness, thereby affecting oxidative stress and cell death. METTL1-mediated m^7^G stabilization of PFKFB3 enhances glycolysis and radioresistance in esophageal cancer (Xiao et al., 2025). NAT10-related programs support metabolic remodeling in lung adenocarcinoma through the GSDMC-CAMKK2-AMPK axis and may also connect ac^4^C-dependent translation to glycolysis and immune suppression (Chen et al., 2023). m^6^A and m^5^C regulators can shape lactate, lipid metabolism, and ferroptosis-related pathways, linking metabolism with both tumor-intrinsic resistance and the immune microenvironment (Zhang J. et al., 2026; Dai et al., 2026; Dong et al., 2021; Jiang et al., 2025; Wen et al., 2024). Synthesis and open questions. Stemness and metabolism should be considered adaptive states rather than separate endpoints. RNA modifications may allow resistant clones to couple repair, antioxidant defense, and immune evasion. Future studies should use lineage tracing, single-cell profiling, and temporal epitranscriptomic mapping to test whether fractionated RT selects pre-existing RNA modification-defined resistant clones or induces new adaptive RNA modification states.

## 4 RNA modifications shape the tumor microenvironment during RT

**FIGURE 2:**
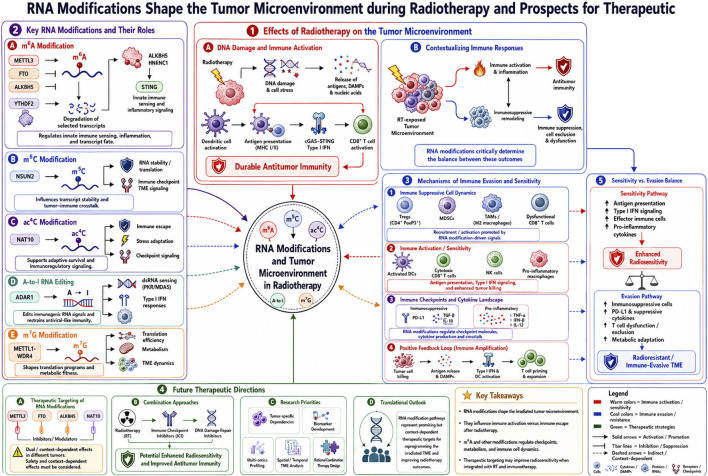
RNA modifications shape the tumor microenvironment during radiotherapy and provide therapeutic opportunities. RT can activate antitumor immunity through antigen release, cGAS-STING signaling, type I interferon responses, antigen presentation, and CD8^+^ T-cell activation. RNA modifications can either strengthen these responses or promote immune evasion by regulating immune checkpoints, cytokines, suppressive myeloid cells, metabolic adaptation, and antigen presentation. The figure also summarizes potential therapeutic strategies, including targeting RNA modification pathways together with RT, immune checkpoint blockade, DNA damage repair inhibitors, or metabolic directed therapies.

RT can convert local tumor injury into systemic antitumor immunity by releasing antigens, activating cGAS-STING signaling, increasing type I interferon responses, and promoting dendritic-cell and CD8^+^ T-cell activation (Hsieh et al., 2022; King et al., 2017). However, the irradiated tumor microenvironment can also become immunosuppressive, with increased immune checkpoints, suppressive cytokines, myeloid recruitment, and T-cell exhaustion. RNA modifications regulate this activation-versus-evasion balance, as summarized in Figure 2.

### 4.1 Innate immune sensing and antigen presentation

Radiation-induced nucleic acid sensing is an important entry point for radioimmunity. m^6^A can fine-tune danger-signal transcripts after irradiation. ALKBH5 removes m^6^A from the 3′UTR of HMGB1 mRNA, increasing HMGB1 expression and activating STING-IRF3 signaling, whereas YTHDF2 promotes degradation of m6A-modified HMGB1 (Chatterjee and Walker, 2017). This example illustrates how erasers and readers can exert opposite effects on the same transcript. ADAR1 provides a parallel non-m^6^A mechanism: by editing endogenous dsRNA, ADAR1 limits MDA5/PKR activation and type I interferon signaling (Song et al., 2022; Ishizuka et al., 2019). ADAR1 loss can overcome resistance to PD-1 blockade in tumors with defective antigen presentation, supporting its potential relevance to radioimmunotherapy (Ishizuka et al., 2019). Antigen presentation is also regulated by m^6^A readers. Tumor-intrinsic YTHDF1 deficiency reduces translation of lysosomal genes, limits antigen degradation, and improves response to immune checkpoint blockade (Lin et al., 2023). In RT models, YTHDF2 is induced in dendritic cells and mediates m^6^A-dependent degradation of Notch pathway regulators, impairing MHC-I cross-presentation and CD8^+^ T-cell activation (Chen et al., 2026). Thus, RNA modifications can act in tumor cells and antigen-presenting cells to determine whether RT-induced antigen release becomes effective T-cell priming.

### 4.2 Immune checkpoints and immunosuppressive niches

RNA modifications regulate immune checkpoints in a cancer- and reader-dependent manner. In gastric cancer, METTL3 installs m^6^A marks in the 3′UTR of PDL1 mRNA and promotes YTHDF2-dependent degradation, so METTL3 inhibition can increase PD-L1 expression and improve anti-PD-1 efficacy (Tang et al., 2023). In contrast, in glioblastoma, METTL14 stabilizes PD-L1 mRNA through IGF2BP2 and promotes immune escape (Zhang Z. et al., 2026). ALKBH5 can recruit PD-L1+ macrophages through the MAP3K8-JNK/ERK-IL-8 axis in hepatocellular carcinoma (You et al., 2022). Ac^4^C also intersects with immune checkpoints: NAT10-mediated ac^4^C regulation of KPNB1 promotes PD-L1 nuclear translocation and radioresistance in non-small cell lung cancer (Zhu et al., 2025). These findings argue against a simple model in which one RNA-modifying enzyme uniformly increases or decreases PD-L1 (Li et al., 2025b). RNA modifications also shape myeloid and metabolic immunosuppression. METTL3 promotes JAK1-STAT3 signaling in tumor-associated macrophages, and lactate can increase METTL3 activity through histone lactylation (Dong et al., 2021). Hypoxia-induced ALKBH5 stabilizes NEAT1 and promotes CXCL8-dependent TAM recruitment (Dong et al., 2021). METTL3 and YTHDF1 can enhance CXCL1-related MDSC migration through BHLHE41 or p65-dependent pathways (Xiong et al., 2022; Bao et al., 2023). Non-m^6^A marks participate as well: NSUN2-mediated m^5^C can regulate metabolic genes such as SOAT2 and weaken CD8^+^ T-cell activity (Jiang et al., 2025), while METTL1-mediated m^7^G can enhance PKM2 and immunosuppressive CD155 signaling (Wen et al., 2024). Figure 2 integrates these immune-cell, checkpoint, cytokine, and metabolic pathways.

RNA modifications should be viewed as one regulatory layer within a broader RT-conditioned immune ecosystem, not as isolated immune switches (Li et al., 2023). Their net effect depends on cell type, timing, and dose pattern. A key challenge is to determine whether targeting an RNA-modifying factor enhances antitumor immunity, reduces normal-tissue inflammation, or produces both effects in different compartments. Spatial transcriptomics and single-cell epitranscriptomic approaches will be essential for separating tumor-cell, immune-cell, stromal, and normal-tissue responses after RT.

## 5 Translational implications and barriers to clinical application

A focused translational framework should prioritize target selection, rational combinations, biomarkers, delivery, and safety rather than repeating the mechanisms described above. Candidate interventions are most compelling when an RNA-modifying factor has a defined tumor-specific dependency, a measurable biomarker, and a plausible therapeutic window.

### 5.1 Candidate targets and rational combinations

METTL3 is among the most druggable RNA-modifying enzymes. The catalytic inhibitor STM2457 reduces m^6^A on leukemia-related transcripts, induces differentiation and apoptosis, impairs leukemia engraftment, and targets stem-cell populations in AML models (Yankova et al., 2021). For RT combinations, METTL3 inhibition could be explored in tumors where METTL3 supports repair, stemness, or immune evasion, but PD-L1 regulation by METTL3 is context dependent and should be biomarker guided (Xu et al., 2023; Du et al., 2023; Visvanathan et al., 2018; Huang et al., 2024; Fang M. et al., 2025). FTO inhibitors may reduce stemness, DNA repair capacity, and immune escape, with preclinical evidence from leukemia and glioblastoma models (Zhang J. et al., 2025; Su et al., 2020). ALKBH5 targeting requires particular caution because ALKBH5 can either enhance inflammatory sensing or support immunosuppressive macrophage recruitment depending on context (Chen et al., 2021; You et al., 2022). Non-m^6^A targets are also plausible but less mature. NAT10 inhibition may affect ac^4^C-dependent translation, PD-L1 localization, checkpoint recovery, and glycolytic immune suppression (Liu et al., 2020; Zhu et al., 2025; Chen et al., 2023). ADAR1 inhibition could amplify dsRNA sensing and type I interferon signaling, potentially enhancing RT plus immune checkpoint blockade (Ishizuka et al., 2019; Roulois et al., 2015). METTL1-WDR4 or NSUN-family targeting may alter glycolysis, translation, homologous recombination, or ferroptosis resistance (Zheng et al., 2026; Xiao et al., 2025; Yu et al., 2024; Wen et al., 2024). These strategies may be most effective in combinations with RT, immune checkpoint inhibitors, DNA repair inhibitors such as PARP/ATR/CHK1/DNA-PK inhibitors, or ferroptosis inducers (Sharma et al., 2025; Wu et al., 2024).

### 5.2 Barriers to clinical translation

Despite the strong biological rationale for targeting RNA modification pathways to enhance RT response, clinical translation remains at an early stage. First, there is currently a lack of clinical-stage inhibitors that have been specifically validated in combination with RT. Although inhibitors targeting METTL3, FTO, ALKBH5, NAT10, or ADAR1-related pathways have shown activity in preclinical cancer models, most have not yet been tested in prospective RT-based clinical trials. Therefore, their optimal dose, treatment sequence, radiosensitizing efficacy, and safety profile in irradiated patients remain unclear. Second, potential normal tissue toxicity represents a major concern. RNA-modifying enzymes are not tumor-specific factors; they also regulate normal cell homeostasis, stem/progenitor cell renewal, immune-cell function, epithelial repair, and stress responses. Systemic inhibition may therefore increase radiation injury in radiosensitive tissues, including bone marrow, intestinal crypts, skin, lung, and immune compartments. This is particularly important because the same RNA modification pathway that promotes tumor radioresistance may also support normal tissue recovery after irradiation. Third, tumor-specific delivery strategies will likely be required to improve the therapeutic window. Nanoparticles, antibody-drug conjugates, tumor-targeted RNA therapeutics, or locally delivered formulations may help restrict RNA modification-targeted interventions to tumor tissues and reduce systemic toxicity. However, these approaches remain technically challenging and require rigorous pharmacokinetic and biodistribution evaluation. Fourth, predictive biomarkers are still insufficiently validated. Most current studies rely on expression changes of individual writers, erasers, or readers in cell lines or small retrospective cohorts. Robust patient-derived biomarkers are needed to identify tumors that are truly dependent on specific RNA modification pathways. Future clinical translation will require integration of RNA modification profiles, transcriptomic signatures, immune contexture, tumor heterogeneity, and RT dose-response data in well-annotated patient cohorts.

Several key limitations should be considered when interpreting non-m^6^A modifications in RT response. First, global radiation-induced changes in m^5^C, ac^4^C, m^7^G, and A-to-I editing have not been systematically mapped in most tumor models. As a result, it remains difficult to distinguish broad epitranscriptomic remodeling from isolated target-specific events. Second, many regulators of these modifications may have modification-independent functions. For example, NAT10 can regulate DNA damage checkpoints through protein acetylation, and ADAR1 may influence DNA repair and innate immune signaling beyond its editing-dependent activity (Xie R. et al., 2023). Third, potential crosstalk between non-m^6^A modifications and m^6^A remains largely unexplored. Different marks may coexist on the same transcript or regulate convergent pathways such as DNA repair, ferroptosis, metabolism, and immune escape. Future studies should therefore combine global profiling, catalytic-mutant rescue experiments, and multi-modification mapping to determine whether non-m^6^A marks act independently, cooperate with m^6^A, or compensate for m^6^A-dependent regulatory programs after irradiation.

## 6 Discussion

Most current studies evaluate RNA modifications after a single radiation dose or at one fixed time point. However, clinical RT is commonly delivered as fractionated treatment, in which tumor and normal cells experience repeated cycles of DNA damage, repair, oxidative stress, inflammation, and adaptive recovery. Fractionated RT may therefore induce RNA modification programs that differ from those triggered by single high-dose irradiation. For example, repeated irradiation may gradually select resistant clones with stable epitranscriptomic states, whereas single-dose irradiation may mainly capture acute stress-induced modification changes. Dynamic profiling across different doses, time points, and fractionation schedules is needed to distinguish transient RNA modification responses from durable programs that drive acquired radioresistance. Future studies should directly compare single-dose and fractionated RT models using MeRIP-seq, RNA bisulfite sequencing, acRIP-seq, RNA editing profiling, and Ribo-seq to determine whether specific RNA modification signatures predict adaptive resistance or radiosensitization.

Non-coding RNAs also represent an important layer of epitranscriptomic regulation in radioresistance. Circular RNAs and long non-coding RNAs can act either as direct substrates of RNA modifications or as regulators that recruit, scaffold, or sequester RNA-modifying enzymes and reader proteins (Zhang et al., 2023; Qin et al., 2021). For example, m^6^A- or m^5^C-modified circRNAs and lncRNAs may influence RNA stability, nuclear export, translation, miRNA sponging, DNA damage repair, immune signaling, and stemness-related pathways. In some cases, modified non-coding RNAs may serve as molecular bridges between RNA modification machinery and classical radioresistance pathways, such as PARP1-dependent DNA repair, RAD51-mediated homologous recombination (Tsang and Munster, 2022), ferroptosis resistance, or immune checkpoint regulation. However, this field remains fragmented, and most studies still focus on individual circRNA/lncRNA axes. Future work should systematically map modification sites on non-coding RNAs before and after irradiation and determine whether these modified transcripts function as drivers, biomarkers, or byproducts of radioresistance.

## 7 Future perspective

Future studies should move beyond descriptive associations between RNA modification factors and radioresistance and instead test specific, RT-relevant hypotheses. First, it remains unclear whether fractionated RT induces RNA modification signatures that differ from those triggered by single high-dose irradiation. Dynamic profiling of m^6^A, m^5^C, ac^4^C, m^7^G, and A-to-I editing across dose fractions and time points may help distinguish acute stress responses from stable epitranscriptomic programs that drive acquired radioresistance. Second, future work should determine whether radiosensitive and radioresistant tumor clones exhibit distinct RNA modification landscapes. Single-cell sequencing combined with MeRIP-seq, RNA editing profiling, Ribo-seq, and functional screening could identify modification-dependent survival programs in resistant subpopulations, including cancer stem-like cells and hypoxic tumor cells. Third, spatial mapping of RNA modification regulators in irradiated tumors is needed to define how epitranscriptomic states vary across tumor, stromal, immune, and normal tissue compartments. This is particularly important for understanding whether RNA modifications promote radiation-induced antitumor immunity or immunosuppressive remodeling. Fourth, therapeutic hypotheses should be tested in clinically relevant combination models. For example, YTHDF1 or YTHDF2 inhibition may improve antigen presentation and enhance RT combined with immune checkpoint blockade. Similarly, targeting METTL3, FTO, ALKBH5, NAT10, or ADAR1 may increase radiosensitivity only in selected tumor contexts with validated pathway dependence.

## References

[B1] An Y. Duan H. (2022). The role of m6A RNA methylation in cancer metabolism. Mol. Cancer 21, 14. 10.1186/s12943-022-01500-4 35022030 PMC8753874

[B2] Bao Y. Zhai J. Chen H. Wong C. C. Liang C. Ding Y. (2023). Targeting m(6)A reader YTHDF1 augments antitumour immunity and boosts anti-PD-1 efficacy in colorectal cancer. Gut 72, 1497–1509. 10.1136/gutjnl-2022-328845 36717220 PMC10359538

[B3] Biserova K. Jakovlevs A. Uljanovs R. Strumfa I. (2021). Cancer stem cells: significance in origin, pathogenesis and treatment of glioblastoma. Cells 10 (3), 621. 10.3390/cells10030621 33799798 PMC8000844

[B4] Chatterjee N. Walker G. C. (2017). Mechanisms of DNA damage, repair, and mutagenesis. Environ. Molecular Mutagenesis 58, 235–263. 10.1002/em.22087 28485537 PMC5474181

[B5] Chellamuthu A. Gray S. G. (2020). The RNA methyltransferase NSUN2 and its potential roles in cancer. Cells 9 (8), 1758. 10.3390/cells9081758 32708015 PMC7463552

[B6] Chen G. Zhao Q. Yuan B. Wang B. Zhang Y. Li Z. (2021). ALKBH5-Modified HMGB1-STING activation contributes to radiation induced liver disease via innate immune response. Int. Journal Radiation Oncology, Biology, Physics 111, 491–501. 10.1016/j.ijrobp.2021.05.115 34044094

[B7] Chen X. Hao Y. Liu Y. Zhong S. You Y. Ao K. (2023). NAT10/ac4C/FOXP1 promotes malignant progression and facilitates immunosuppression by reprogramming glycolytic metabolism in cervical cancer. Adv. Science Weinheim, Baden-Wurttemberg, Ger. 10, e2302705. 10.1002/advs.202302705 PMC10646273 37818745

[B8] Chen D. Gu X. Nurzat Y. Xu L. Li X. Wu L. (2024). Writers, readers, and erasers RNA modifications and drug resistance in cancer. Mol. Cancer 23, 178. 10.1186/s12943-024-02089-6 39215288 PMC11363509

[B9] Chen D. Wang L. Wen C. Piffko A. Bugno J. Yu X. (2026). Radiotherapy induces YTHDF2 in dendritic cells impairing cross-presentation and T cell function. J. Experimental Medicine 223, e20250641. 10.1084/jem.20250641 PMC12924711 41196643

[B10] Dai Z. Lin B. Qin M. Lin Y. Wang L. Liao K. (2025). METTL3-mediated m6A modification of SLC7A11 enhances nasopharyngeal carcinoma radioresistance by inhibiting ferroptosis. Int. Journal Biological Sciences 21, 1837–1851. 10.7150/ijbs.100518 PMC11844296 39990661

[B11] Dai B. Li J. Xu L. Chen W. Chen J. Song M. (2026). YTHDF2-mediated stabilization of SREBF1 promotes lipid metabolic reprogramming and ferroptosis-associated radioresistance in Anaplastic thyroid carcinoma. Cancer Letters 639, 218232. 10.1016/j.canlet.2025.218232 41435981

[B12] Dong F. Qin X. Wang B. Li Q. Hu J. Cheng X. (2021). ALKBH5 facilitates hypoxia-induced paraspeckle assembly and IL8 secretion to generate an immunosuppressive tumor microenvironment. Cancer Research 81, 5876–5888. 10.1158/0008-5472.can-21-1456 34670781

[B13] Du H. Zou N. Y. Zuo H. L. Zhang X. Y. Zhu S. C. (2023). YTHDF3 mediates HNF1α regulation of cervical cancer radio-resistance by promoting RAD51D translation in an m6A-dependent manner. FEBS Journal 290, 1920–1935. 10.1111/febs.16681 36380687

[B14] Fang Y. Shang S. Chen G. Chen D. Yu J. (2025a). YTHDF2 alleviates the radioresistance of rectal cancer cells by targeting methylated MYC. J. Radiation Research 66, 459–472. 10.1093/jrr/rraf043 PMC12460049 40716096

[B15] Fang M. Li Y. Wang P. Wang Y. Wang X. Wa X. (2025b). METTL3 inhibition restores PD-L1 expression and CD8+ T-cell cytotoxic function in immunotherapy-treated gastric cancer. Cancer Immunology Research 13, 1037–1052. 10.1158/2326-6066.CIR-24-1179 40299705

[B16] Gan W. L. Chen L. (2025). A-to-I RNA editing in hematologic immunity and malignancy. Exp. Hematology 150, 104861. 10.1016/j.exphem.2025.104861 40738341

[B17] Giraud N. Ortholan C. Quivrin M. Andraud M. Cordoba A. Shaffer R. (2025). Radiotherapy: beyond cancer. Cancer radiotherapie J. de la Soc. francaise de radiotherapie Oncol. 29, 104682. 10.1016/j.canrad.2025.104682 40737738

[B18] Gu Z. Zou L. Pan X. Yu Y. Liu Y. Zhang Z. (2024). The role and mechanism of NAT10-mediated ac4C modification in tumor development and progression. MedComm 5, e70026. 10.1002/mco2.70026 39640362 PMC11617596

[B19] Hsieh R. C. Krishnan S. Wu R. C. Boda A. R. Liu A. Winkler M. (2022). ATR-Mediated CD47 and PD-L1 up-regulation restricts radiotherapy-induced immune priming and abscopal responses in colorectal cancer. Sci. Immunology 7, eabl9330. 10.1126/sciimmunol.abl9330 35687697 PMC9373855

[B20] Hu Y. Shan Z. Sun X. Liang J. Wang L. Zhou X. (2026). NSUN2/ALYREF stabilizes m5C-modified circCEP70 to enhance radioresistance in rectal cancer through NEDD8-dependent neddylation and ubiquitination regulation. Int. Journal Biological Macromolecules 366, 152388. 10.1016/j.ijbiomac.2026.152388 42106046

[B21] Huang W. M. Li Z. X. Wu Y. H. Shi Z. L. Mi J. L. Hu K. (2023). m6A demethylase FTO renders radioresistance of nasopharyngeal carcinoma via promoting OTUB1-mediated anti-ferroptosis. Transl. Oncology 27, 101576. 10.1016/j.tranon.2022.101576 PMC9646990 36343416

[B22] Huang J. Li H. Yang Z. Liu R. Li Y. Hu Y. (2024). SALL4 promotes cancer stem-like cell phenotype and radioresistance in oral squamous cell carcinomas via methyltransferase-like 3-mediated m6A modification. Cell Death and Disease 15, 139. 10.1038/s41419-024-06533-9 38355684 PMC10866932

[B23] Ishizuka J. J. Manguso R. T. Cheruiyot C. K. Bi K. Panda A. Iracheta-Vellve A. (2019). Loss of ADAR1 in tumours overcomes resistance to immune checkpoint blockade. Nature 565, 43–48. 10.1038/s41586-018-0768-9 30559380 PMC7241251

[B24] Jhunjhunwala S. Hammer C. Delamarre L. (2021). Antigen presentation in cancer: insights into tumour immunogenicity and immune evasion. Nat. Reviews. Cancer 21, 298–312. 10.1038/s41568-021-00339-z 33750922

[B25] Jiang J. Liu F. Cui D. Xu C. Chi J. Yan T. (2025). Novel molecular mechanisms of immune evasion in hepatocellular carcinoma: NSUN2-Mediated increase of SOAT2 RNA methylation. Cancer Communications Lond. Engl. 45, 846–879. 10.1002/cac2.70023 PMC12328098 40227950

[B26] King K. R. Aguirre A. D. Ye Y. X. Sun Y. Roh J. D. Ng R. P. Jr. (2017). IRF3 and type I interferons fuel a fatal response to myocardial infarction. Nat. Medicine 23, 1481–1487. 10.1038/nm.4428 29106401 PMC6477926

[B27] Kowalski-Chauvel A. Lacore M. G. Arnauduc F. Delmas C. Toulas C. Cohen-Jonathan-Moyal E. (2020). The m6A RNA demethylase ALKBH5 promotes radioresistance and invasion capability of glioma stem cells. Cancers 13, 40. 10.3390/cancers13010040 33375621 PMC7795604

[B28] Li P. Huang D. (2024). NSUN2-mediated RNA methylation: molecular mechanisms and clinical relevance in cancer. Cell. Signalling 123, 111375. 10.1016/j.cellsig.2024.111375 39218271

[B29] Li H. S. Liu C. M. Wang Y. (2023). RANKL acts an unfavorable prognostic biomarker and potential target in advanced KRAS-Mutated lung adenocarcinoma. Thorac. Cancer 14, 1368–1382. 10.1111/1759-7714.14882 37021520 PMC10212663

[B30] Li H. S. Tang R. Shi H. S. Qin Z. J. Zhang X. Y. Sun Y. F. (2025a). Ultra-high dose rate radiotherapy overcomes radioresistance in head and neck squamous cell carcinoma. Signal Transduction Targeted Therapy 10, 82. 10.1038/s41392-025-02184-0 40032871 PMC11876629

[B31] Li H. S. Liu C. M. Zheng S. F. Wu P. Xu H. Y. Hao X. Z. (2025b). RANKL/PD-1 dual blockade demonstrates survival benefit for patients with advanced lung adenocarcinoma harboring KRAS mutations. Cell Reports. Med. 6, 102235. 10.1016/j.xcrm.2025.102235 PMC12281428 40669444

[B32] Liang Z. Walkley C. R. Heraud-Farlow J. E. (2024). A-to-I RNA editing and hematopoiesis. Exp. Hematology 139, 104621. 10.1016/j.exphem.2024.104621 39187172

[B33] Lin W. Chen L. Zhang H. Qiu X. Huang Q. Wan F. (2023). Tumor-intrinsic YTHDF1 drives immune evasion and resistance to immune checkpoint inhibitors via promoting MHC-I degradation. Nat. Communications 14, 265. 10.1038/s41467-022-35710-7 PMC9845301 36650153

[B34] Lin Y. Sun Y. Hou W. Chen X. Zhou F. Xu Q. (2024). FTO-Mediated regulation of m6A methylation is closely related to apoptosis induced by repeated UV irradiation. J. Dermatological Science 114, 124–132. 10.1016/j.jdermsci.2024.01.001 38749796

[B35] Liu H. Y. Liu Y. Y. Yang F. Zhang L. Zhang F. L. Hu X. (2020). Acetylation of MORC2 by NAT10 regulates cell-cycle checkpoint control and resistance to DNA-Damaging chemotherapy and radiotherapy in breast cancer. Nucleic Acids Research 48, 3638–3656. 10.1093/nar/gkaa130 32112098 PMC7144926

[B36] Nguyen P. Shukla S. Liu R. Abbineni G. Smart D. K. (2019). Sirt2 regulates radiation-induced injury. Radiat. Research 191, 398–412. 10.1667/RR15282.1 30835165 PMC8237344

[B37] Orellana E. A. Liu Q. Yankova E. Pirouz M. De Braekeleer E. Zhang W. (2021). METTL1-mediated m(7)G modification of Arg-TCT tRNA drives oncogenic transformation. Mol. Cell 81, 3323–3338.e14. 10.1016/j.molcel.2021.06.031 34352207 PMC8380730

[B38] Qin S. Mao Y. Chen X. Xiao J. Qin Y. Zhao L. (2021). The functional roles, cross-talk and clinical implications of m6A modification and circRNA in hepatocellular carcinoma. Int. Journal Biological Sciences 17, 3059–3079. 10.7150/ijbs.62767 PMC8375232 34421350

[B39] Ren D. Li Y. Lu M. Jiang W. Xu H. Wu L. (2026). Gasdermin C reprograms metabolism through the CAMKK2-AMPK axis to promote lung adenocarcinoma progression and radioresistance. Cell Reports 45, 117427. 10.1016/j.celrep.2026.117427 42176271

[B40] Roulois D. Loo Yau H. Singhania R. Wang Y. Danesh A. Shen S. Y. (2015). DNA-demethylating agents target colorectal cancer cells by inducing viral mimicry by endogenous transcripts. Cell 162, 961–973. 10.1016/j.cell.2015.07.056 26317465 PMC4843502

[B41] Roundtree I. A. Evans M. E. Pan T. He C. (2017). Dynamic RNA modifications in gene expression regulation. Cell 169, 1187–1200. 10.1016/j.cell.2017.05.045 28622506 PMC5657247

[B42] Sharma R. Mishra A. Bhardwaj M. Singh G. Indira Harahap L. V. Vanjani S. (2025). Medicinal chemistry breakthroughs on ATM, ATR, and DNA-PK inhibitors as prospective cancer therapeutics. J. Enzyme Inhibition Medicinal Chemistry 40, 2489720. 10.1080/14756366.2025.2489720 PMC12013171 40256842

[B43] Shen Y. Liu W. Zhou Z. He J. Qi X. (2025). FTO-Mediated m6A Demethylation of OTUB1 stabilizes SLC7A11 to alleviate ferroptosis in cerebral ischemia/reperfusion injury. J. Stroke Cerebrovascular Diseases The Official Journal Natl. Stroke Assoc. 34, 108316. 10.1016/j.jstrokecerebrovasdis.2025.108316 40233842

[B44] Shi X. Zhang X. Huang X. Zhang R. Pan S. Huang S. (2024). N(6)-methyladenosine-mediated upregulation of LNCAROD confers radioresistance in esophageal squamous cell carcinoma through stabilizing PARP1. Clin. Translational Medicine 14, e70039. 10.1002/ctm2.70039 PMC11452732 39367700

[B45] Song B. Shiromoto Y. Minakuchi M. Nishikura K. (2022). The role of RNA editing enzyme ADAR1 in human disease. Wiley Interdisciplinary Reviews. RNA 13, e1665. 10.1002/wrna.1665 34105255 PMC8651834

[B46] Su R. Dong L. Li Y. Gao M. Han L. Wunderlich M. (2020). Targeting FTO suppresses cancer stem cell maintenance and immune evasion. Cancer Cell 38, 79–96.e11. 10.1016/j.ccell.2020.04.017 32531268 PMC7363590

[B47] Sun H. Li K. Liu C. Yi C. (2023). Regulation and functions of non-m(6)A mRNA modifications. Nat. Reviews. Mol. Cell Biology 24, 714–731. 10.1038/s41580-023-00622-x 37369853

[B48] Tang Z. Sun C. Yan Y. Niu Z. Li Y. Xu X. (2023). Aberrant elevation of FTO levels promotes liver steatosis by decreasing the m6A methylation and increasing the stability of SREBF1 and ChREBP mRNAs. J. Molecular Cell Biology 14, mjac061. 10.1093/jmcb/mjac061 PMC9951264 36352530

[B49] Tang R. Yin J. Liu Y. Xue J. (2024). FLASH radiotherapy: a new milestone in the field of cancer radiotherapy. Cancer Letters 587, 216651. 10.1016/j.canlet.2024.216651 38342233

[B50] Tian C. Li C. Wang J. Liu Y. Gao J. Hong X. (2025). ADAR1 enhances tumor proliferation and radioresistance in non-small cell lung cancer by interacting with Rad18. Cell. Oncology Dordr. Neth. 48, 471–485. 10.1007/s13402-024-01012-x PMC11996937 39570561

[B51] Tsang E. S. Munster P. N. (2022). Targeting RAD51-Mediated homologous recombination as a treatment for advanced solid and hematologic malignancies: opportunities and challenges ahead. OncoTargets Therapy 15, 1509–1518. 10.2147/ott.s322297 36536949 PMC9758980

[B52] Visvanathan A. Patil V. Arora A. Hegde A. S. Arivazhagan A. Santosh V. (2018). Essential role of METTL3-mediated m(6)A modification in glioma stem-like cells maintenance and radioresistance. Oncogene 37, 522–533. 10.1038/onc.2017.351 28991227

[B53] Vozenin M. C. Bourhis J. Durante M. (2022). Towards clinical translation of FLASH radiotherapy. Nat. Reviews. Clin. Oncology 19, 791–803. 10.1038/s41571-022-00697-z 36303024

[B54] Wan W. Ao X. Chen Q. Yu Y. Ao L. Xing W. (2022). METTL3/IGF2BP3 axis inhibits tumor immune surveillance by upregulating N(6)-methyladenosine modification of PD-L1 mRNA in breast cancer. Mol. Cancer 21, 60. 10.1186/s12943-021-01447-y 35197058 PMC8864846

[B55] Wang H. Chen W. Cui Y. Gong H. Li H. (2023a). KIAA1429 protects hepatocellular carcinoma cells from ferroptotic cell death with a m(6) A-dependent posttranscriptional modification of SLC7A11. J. Cellular Molecular Medicine 27, 4118–4132. 10.1111/jcmm.17997 PMC10746954 37830241

[B56] Wang Y. Zhang L. Sun X. L. Lu Y. C. Chen S. Pei D. S. (2023b). NRP1 contributes to stemness and potentiates radioresistance via WTAP-Mediated m6A methylation of Bcl-2 mRNA in breast cancer. Apoptosis. An International Journal Programmed Cell Death 28, 233–246. 10.1007/s10495-022-01784-3 36333630

[B57] Wang B. Zhang Y. Niu H. Zhao X. Chen G. Zhao Q. (2024). METTL3-Mediated STING upregulation and activation in kupffer cells contribute to radiation-induced liver disease via pyroptosis. Int. Journal Radiation Oncology, Biology, Physics 119, 219–233. 10.1016/j.ijrobp.2023.10.041 37914138

[B58] Wen J. Xue L. Wei Y. Liang J. Jia W. Yong T. (2024). YTHDF2 is a therapeutic target for HCC by suppressing immune evasion and angiogenesis through ETV5/PD-L1/VEGFA axis. Adv. Science Weinheim, Baden-Wurttemberg, Ger. 11, e2307242. 10.1002/advs.202307242 PMC10987122 38247171

[B59] Wen C. Naccasha E. Z. He C. Liang H. L. Weichselbaum R. R. (2025). YTHDFs as radiotherapy checkpoints in tumor immunity. J. Experimental Medicine 222, e20250272. 10.1084/jem.20250272 PMC12139520 40471119

[B60] Wu Y. Song Y. Wang R. Wang T. (2023). Molecular mechanisms of tumor resistance to radiotherapy. Mol. Cancer 22, 96. 10.1186/s12943-023-01801-2 37322433 PMC10268375

[B61] Wu D. Spencer C. B. Ortoga L. Zhang H. Miao C. (2024). Histone lactylation-regulated METTL3 promotes ferroptosis via m6A-modification on ACSL4 in sepsis-associated lung injury. Redox Biology 74, 103194. 10.1016/j.redox.2024.103194 38852200 PMC11219935

[B62] Xiang Y. Laurent B. Hsu C. H. Nachtergaele S. Lu Z. Sheng W. (2017). RNA m(6)A methylation regulates the ultraviolet-induced DNA damage response. Nature 543, 573–576. 10.1038/nature21671 28297716 PMC5490984

[B63] Xiao C. Hou G. Wang C. Huang Y. Liu Z. (2025). METTL1 mediates m7G modification of PFKFB3 mRNA to promote radioresistance in esophageal cancer by affecting glycolytic metabolism. Pathology, Research Practice 272, 156102. 10.1016/j.prp.2025.156102 40633178

[B64] Xie L. Zhong X. Cao W. Liu J. Zu X. Chen L. (2023a). Mechanisms of NAT10 as ac4C writer in diseases. Molecular therapy. Nucleic Acids. 32, 359–368. 10.1016/j.omtn.2023.03.023 37128278 PMC10148080

[B65] Xie R. Cheng L. Huang M. Huang L. Chen Z. Zhang Q. (2023b). NAT10 drives cisplatin chemoresistance by enhancing ac4C-Associated DNA repair in bladder cancer. Cancer Research 83, 1666–1683. 10.1158/0008-5472.can-22-2233 36939377

[B66] Xiong J. He J. Zhu J. Pan J. Liao W. Ye H. (2022). Lactylation-driven METTL3-mediated RNA m(6)A modification promotes immunosuppression of tumor-infiltrating myeloid cells. Mol. Cell 82, 1660–1677.e10. 10.1016/j.molcel.2022.02.033 35320754

[B67] Xu X. Zhang P. Huang Y. Shi W. Mao J. Ma N. (2023). METTL3-mediated m6A mRNA contributes to the resistance of carbon-ion radiotherapy in non-small-cell lung cancer. Cancer Science 114, 105–114. 10.1111/cas.15590 36114749 PMC9807515

[B68] Yang Z. Yang S. Cui Y. H. Wei J. Shah P. Park G. (2021). “METTL14 facilitates global genome repair and suppresses skin tumorigenesis,” in Proceedings of the National Academy of Sciences of the United States of America, 118. 10.1073/pnas.2025948118 PMC8536359 34452996

[B69] Yankova E. Blackaby W. Albertella M. Rak J. De Braekeleer E. Tsagkogeorga G. (2021). Small-molecule inhibition of METTL3 as a strategy against myeloid leukaemia. Nature 593, 597–601. 10.1038/s41586-021-03536-w 33902106 PMC7613134

[B70] You Y. Wen D. Zeng L. Lu J. Xiao X. Chen Y. (2022). ALKBH5/MAP3K8 axis regulates PD-L1+ macrophage infiltration and promotes hepatocellular carcinoma progression. Int. Journal Biological Sciences 18, 5001–5018. 10.7150/ijbs.70149 PMC9379398 35982895

[B71] Yu M. Ni M. Xu F. Liu C. Chen L. Li J. (2024). NSUN6-mediated 5-methylcytosine modification of NDRG1 mRNA promotes radioresistance in cervical cancer. Mol. Cancer 23, 139. 10.1186/s12943-024-02055-2 38970106 PMC11225205

[B72] Zhang Q. Wei T. Yan L. Zhu S. Jin W. Bai Y. (2023). Hypoxia-responsive lncRNA AC115619 encodes a micropeptide that suppresses m6A modifications and hepatocellular carcinoma progression. Cancer Research 83, 2496–2512. 10.1158/0008-5472.can-23-0337 37326474

[B73] Zhang Y. Li L. Mendoza J. J. Wang D. Yan Q. Shi L. (2024). Advances in A-to-I RNA editing in cancer. Mol. Cancer 23, 280. 10.1186/s12943-024-02194-6 39731127 PMC11673720

[B74] Zhang C. Yang T. Chen H. Ding X. Chen H. Liang Z. (2025a). METTL3 inhibition promotes radiosensitivity in hepatocellular carcinoma through regulation of SLC7A11 expression. Cell Death and Disease 16, 9. 10.1038/s41419-024-07317-x 39799112 PMC11724875

[B75] Zhang J. Li G. Wu R. Shi L. Tian C. Jiang H. (2025b). The m6A RNA demethylase FTO promotes radioresistance and stemness maintenance of glioma stem cells. Cell. Signalling 132, 111782. 10.1016/j.cellsig.2025.111782 40185350

[B76] Zhang L. Wei J. Zou Z. He C. (2026a). RNA modification systems as therapeutic targets. Nat. Reviews. Drug Discovery 25, 59–78. 10.1038/s41573-025-01280-8 40962853

[B77] Zhang J. Shen J. Zeng T. Gao C. Sheng B. Li J. (2026b). Targeting the NSUN2-DHODH axis reverses ferroptosis resistance and oxaliplatin resistance in colorectal cancer. Front. Pharmacology 17, 1739981. 10.3389/fphar.2026.1739981 PMC12968206 41808866

[B78] Zhang Z. Guo X. Qi T. Wang C. Zhai X. Wang M. (2026c). METTL14/IGF2BP2-mediated m6A modification of PD-L1 promotes proliferation, metastasis, and immune escape in high-grade gliomas. J. Neuropathology Experimental Neurology 85, 39–49. 10.1093/jnen/nlaf090 40796110

[B79] Zheng L. Li M. Li X. Wei J. Xue C. Wei Q. (2026). A novel small-molecule inhibitor GSK-F1 confers radiosensitivity by inhibiting the NSUN2/TP53/RAD51 axis-mediated DNA homologous recombination repair in nasopharyngeal carcinoma. Int. Journal Biological Sciences 22, 4043–4058. 10.7150/ijbs.130087 PMC13137861 42088430

[B80] Zhou X. Xia Q. Wang B. Li J. Liu B. Wang S. (2025). USP14 modulates stem-like properties, tumorigenicity, and radiotherapy resistance in glioblastoma stem cells through stabilization of MST4-phosphorylated ALKBH5. Theranostics 15, 2293–2314. 10.7150/thno.103629 39990235 PMC11840735

[B81] Zhou Q. Li C. Jiang X. Yuan Y. Zhou Q. Wang Q. (2026). NSUN5 and RNA m(5)C epitranscriptomic regulation in tumor progression. Front. Cell Developmental Biology 14, 1771110. 10.3389/fcell.2026.1771110 PMC12901338 41695396

[B82] Zhu D. Lu M. Cheng H. (2025). NAT10 promotes radiotherapy resistance in non-small cell lung cancer by regulating KPNB1-mediated PD-L1 nuclear translocation. Open Life Sciences 20, 20251065. 10.1515/biol-2025-1065 40109769 PMC11920766

[B83] Zou Y. Guo S. Wen L. Lv D. Tu J. Liao Y. (2024). Targeting NAT10 inhibits osteosarcoma progression via ATF4/ASNS-mediated asparagine biosynthesis. Cell Reports. Med. 5, 101728. 10.1016/j.xcrm.2024.101728 PMC11525028 39293390

